# Immune Imprinting in the Influenza Ferret Model

**DOI:** 10.3390/vaccines8020173

**Published:** 2020-04-08

**Authors:** Amanda L. Skarlupka, Ted M. Ross

**Affiliations:** 1 Center for Vaccines and Immunology, University of Georgia, Athens, GA 30602, USA; skarlupka@uga.edu; 2 Department of Infectious Diseases, University of Georgia, Athens, GA 30602, USA

**Keywords:** animal model, ferret, pre-immunity, imprint, vaccination, influenza

## Abstract

The initial exposure to influenza virus usually occurs during childhood. This imprinting has long-lasting effects on the immune responses to subsequent infections and vaccinations. Animal models that are used to investigate influenza pathogenesis and vaccination do recapitulate the pre-immune history in the human population. The establishment of influenza pre-immune ferret models is necessary for understanding infection and transmission and for designing efficacious vaccines.

## 1. Introduction

Nearly 500,000 annual global deaths are attributed to influenza virus infection [1]. The influenza virus consists of four different types (A, B, C, D) with Types A and B causing symptoms in humans. These two types are further classified into subtypes and sub-lineages, respectively. The surface of the influenza virion consists of two major glycoproteins the hemagglutinin (HA; Type A: H1-H18; Type B: Yamagata-, Victoria-lineage) and the neuraminidase (NA; Type A: N1-N11), each of which are separated into antigenically distinct subtypes and together denote viral subtypes [2]. The glycoproteins undergo independent evolution leading to antigenic drift as the virus accumulates mutations over time. The HA and NA gene segments reassort due to the segmented nature of the viral RNA genome, leading to antigenic shifts. These shifts are unprecedented and are often met with no neutralizing antibodies in the human population, leading to heightened pandemic potential. Within subtypes, there can be distinct shifts in antigenicity, such as with the seasonal A(H1N1) which circulated in humans until the emergence of the swine-origin 2009 pandemic A(H1N1). The surrogates of protection, induced either through infection or vaccination, rely on serological assays that are dependent on the presence of neutralizing antibodies to mainly the HA glycoprotein: hemagglutination inhibition, single radial hemolysis, and microneutralization [3].

Initial exposure to influenza occurs during childhood; at six to nine years old, 80% of children were seropositive to A(H3N2) [4]. This initial infection primes the immune system and biases future immune responses to subsequent infections and vaccinations [5,6,7,8,9,10,11]. Due to the cost and resources of human clinical trials, there are numerous animal models designed to study this viral pathogen [12]. The ferret model remains indispensable due to similarities in lung physiology [13,14], anatomical distribution of sialylated glycan receptors [15], and glycomic profile of ferret respiratory tissues [16,17]. These animal models are commonly used to study viral characteristics, host immune responses, and vaccine/antiviral therapies. Therefore, animal models that mimic pre-existing human immunity to influenza viruses may better represent the human immune responses to infection and vaccination.

## 2. Influenza Pre-Immunity in the Human Population

The first influenza strain that infects a human or animal is the imprinting virus for that individual [5,18]. This initial strain biases the immune memory response to subsequent infections in a phenomenon described as “original antigenic sin”. The premise is that a when mounting a secondary response to a closely related antigen, instead of creating a completely new and distinct response, the body recalls memory B-cells from within its repertoire of antibodies elicited towards the first virus [19]. This allows for a stronger and faster antibody response, but it may not be as effective as creating a custom response for the second infecting virus. The imprinting strain is associated with the first influenza virus infection. Correlations between the imprinting strain and age of birth results in natural serological groups in human cohorts due to differences in the circulating influenza viral subtypes and strains at the time of infection [5].

The immune responses of differentially imprinted age-groups can be compared to uncover the long-term biases due to differences in pre-immunity. Individuals who were children from 1957 to 1968 were imprinted with circulating A(H2N2) viruses, whereas children born between 1968 to 1977 were likely imprinted with A(H3N2) subtypes. Furthermore, even within the earlier group of children from 1957 and 1968, the children exposed to pandemic A(H2N2) strains had differential neutralization responses to A(H2N2) viruses compared to children exposed to the late seasonal A(H2N2) strains [20]. Hence, antigenic drift contributes to imprinting biases. On a broader scale, the imprinting HA-phylogenetic group (group 1 vs. group 2) is associated with susceptibility to future pandemics [5,21,22,23,24].

Although imprinting was initially characterized by serological results, the basis of this phenomenon is cellular, with the initial infection driving the production of long-lasting virus specific B- and T-memory cells that bias the immune response to subsequence viruses [25]. After this initial infection, pre-existing immunity is the culmination of the first and all following exposures and can also be referred to as an individual’s entire influenza history [18]. A pre-immune individual is one with any immune memory to influenza. Using pre-immune models to determine the dangers of zoonotic transmission, especially from swine-origin, is particularly beneficial [4,26]. During the A(H1N1) 2009 influenza virus pandemic, the different priming patterns in the population contributed to an unprecedented age-biased distribution in human morbidity [27]. All together, these factors highlight the necessity for unraveling the impact of imprinting and pre-immunity on subsequent infection and vaccination.

## 3. Influenza Vaccines, Selection, and Animal Models

The current preventative method used to combat influenza is vaccination. Administered annually, the most common intramuscular split-inactivated cell-based influenza vaccine is composed of four viral strains, an A(H3N2), an A(H1N1), and two type Bs, one each from the Yamagata and Victoria lineages. For the seasons between 2014 and 2019, the adjusted overall vaccine effectiveness (VE) in the United States ranged between 19% and 48% [28,29,30,31,32]. The VE varies between subtypes and year to year due to numerous viral (antigenic drift/shift), vaccine (egg-adaptive changes, immunogenicity) and host (age, immune-status, pre-immunity) factors. To address the viral contributions, the World Health Organization (WHO) reviews data from its global influenza surveillance network to identify circulating strains, heavily weighting ferret serological cross-reactivity data to determine antigenic drifts. Vaccine manufacturers are then provided a list of recommended vaccine strains for that particular season for either the Northern or Southern hemisphere. Methods to increase the host response to vaccination and VE include different vaccine compositions, especially in adults older than 65 years of age due to their decreased immune response to vaccination [33]. The inclusion of adjuvants, varying the amount of antigen, using live-attenuated viral vaccines and varying the route of administration are all methods used to increase VE.

The gold standard for influenza vaccine research is the ferret animal model [34]. Currently, there are models developed that capture infant [35], aged [36], naïve, and pre-immune scenarios [37]. During vaccine selection, experts consider only data generated from the naïve ferret model for determination of recommended strains for currently administered human vaccines. However, the immune response in a naïve host, compared to a pre-immune individual, differs during subsequent vaccination and/or infection [38,39]. Therefore, the use of naïve ferret sera for vaccine strain selection is potentially misrepresentative of the pre-immune human population that receives each season’s influenza virus vaccines, contributing to the observed VE.

## 4. Vaccination as a Surrogate for Viral Infection Pre-Immunity

A pre-immune animal model should be established through an initial viral infection, instead of through a vaccination regimen. Vaccination cannot be a surrogate for viral imprinting and pre-immunity due to the inequivalences in the immune responses to an active influenza infection versus an intramuscular unadjuvanted vaccination [38]. Administering a vaccine matched to the challenge virus does not produce a vaccine with 100% efficacy. Healthy volunteers vaccinated with inactivated or cold-adapted live influenza vaccines were not all protected from challenge with homologous viruses; the estimated protective efficacies were 71% and 85%, respectively [40]. Vaccination does not induce a robust T-cell response compared to infection, which, in ferrets, has been found to contribute to sterilizing immunity [41]. Furthermore in ferrets, vaccination and viral pre-immunity differ in their protective outcomes as well [41]. Significant immunological differences, such as ratios of IgG and IgA influenza-specific antibodies and targeted antigenic sites [26], cannot be discerned and identified with the commonly used hemagglutinin inhibition assay (HAI).

A *pre-immune-vaccination* ferret model was used to investigate the phenomenon of low efficacy from repeat vaccination with commercial quadrivalent inactivated influenza vaccine that occurs in humans [42]. When matched to ferrets vaccinated once, repeatedly vaccinated ferrets had less protection, higher viral shedding and lower T-lymphocyte counts, whereas the serological responses, cell-mediated immunity, and histopathological changes did not differ. It was hypothesized that although the magnitude of the serological response was similar the composition differed, resulting in the difference of protection. A larger ratio of non-neutralizing to neutralizing antibodies may have been recalled in the repeat vaccination group. Hence, the repeat vaccination group had lower vaccine efficacy compared to one vaccination. Encouragingly, the repeat vaccination group was still better protected than no vaccine group. The relevance of using these results to explain the decreased vaccine efficacy in humans is limited due to the lack of pre-immunity establishment in ferrets. Even with well-planned studies, not establishing pre-immunity creates a confounding factor when extrapolating the findings to the human population.

## 5. Low Vaccine Seroconversion Proportions in Naïve Ferrets

In early ferret vaccination studies, the administration of unadjuvanted vaccines elicited no measurable antibody outcome. Not all naïve ferrets seroconvert to influenza vaccination [39,43,44,45]. Even with the addition of an adjuvant, the immune response can be weak, especially when compared to the immune response elicited by a live homologous infection [45,46]. The lack of an antibody response is associated with a lack of protection [47]. For instance, naïve ferrets immunized with A(H3N2)/Hong Kong/X31/1968 vaccine were all susceptible to homologous challenge and none produced vaccine-specific serum HAI antibodies [39]. This low reactivity to the vaccine may be attributed to the outbred nature of the animal model [42].

Low seroconversion ratios may also be due to the low immunogenicity of the influenza vaccine. In humans, vaccines vary in immunogenicity [48,49,50], particularly in immunocompromised adults and children [51]. Due to this issue, pandemic influenza vaccines can be adjuvanted to ensure an efficient immune response [52,53,54,55]. With the inclusion of different adjuvants, different magnitudes of seroconversion and protection can be achieved [47]. Some studies used virus-like particle (VLP) vaccines produced from insect cells using a baculoviral system which results in 100% seroconversion [56,57]. The manufacturing process of these VLPs retain insect protein that act as an adjuvant contributing to seroconversion. Research groups have attempted to solve this phenomenon through multiple vaccinations, i.e., a prime-boost or prime-boost-boost regimen, or with addition of adjuvant to elicit an antibody response [58]. 

Establishment of pre-immunity overcomes this phenomenon; pre-immune ferrets respond to vaccination at a higher proportion than immunologically naïve ferrets. With either type A homosubtypic [59] or heterosubtypic [39] pre-immunity, the vaccine-specific serum hemagglutinin inhibition titers are increased. Imprinting primed the immune system towards future influenza vaccinations. This priming phenomenon is not only present in the ferret animal model, but also occurs in mice and hamsters [60,61,62].

## 6. Historic Pre-Immune Ferret Models

One of the earliest works with pre-immunity in ferrets was conducted by Webster in 1966 investigating the presence of original antigenic sin in ferrets by conducting sequential infections [63]. Following this, in the 1970s, a vast amount of pre-immunity work was conducted with ferrets. These studies focused on characterizing the ferret immune system response to live and killed virus, vaccination, adjuvant and heterotypic and heterologous infections [39,45,46,64,65,66,67]. Further, heterosubtypic immunity was shown not to wane over a period of up to eighteen months [68]. This historical collection of ferret research laid the foundation for showing that low vaccination seroconversion proportions for naïve ferrets can be overcome by the development and optimization of a pre-immune animal model. The pre-immune model never advanced after this time, potentially due to the lack of immunological reagents and tools needed to properly characterize the model and general ignorance of the magnitude imprinting and pre-immunity contributes towards vaccination and infection.

## 7. Current Pre-Immune Ferret Models in Practice

### 7.1. A(H1N1) 2009 Pandemic

After a lull in the pre-immune ferret research, the A(H1N1) 2009 swine influenza pandemic initiated the dramatic increase of the investigative effort into imprinting, pre-immunity, and heterologous protection. The early epidemiological and serological studies that inspired this interest suggested that pre-existing immunity may have altered the pandemic virus’ morbidity and mortality in the human population [9,69]. The resultant pre-immunity models were based upon the historical model: (1) establish anti-influenza virus immune memory with sub-lethal viral challenge; (2) assess for seroconversion; (3) vaccinate, if necessary; (4) challenge with A(H1N1)/California/2009. A prolonged period of rest between imprinting and vaccination or challenge allows the ferret to return to an assumed immunological baseline after the generation of an adaptive memory response and recovery from damage and local cellular activation in the lung tissue. Compared to the 1970s, the drastic increase in the understanding of the immune system, the effects induced from influenza challenge and vaccination, and the ability to measure and quantify these important details allowed for a well-defined model. The worldwide 2009 pandemic inspired much research looking at the protective effects of seasonal A(H1N1) imprinting on the A(H1N1) 2009 pandemic strain. Therefore, much of the published research has focused on the A(H1N1) subtype (Tables S1 and S2).

Sterilizing immunity in ferrets, an immune state that blocks viral infection [41], can be achieved through establishing pre-immunity [70]. Whereas, with an intramuscular vaccination subsequent infection was not inhibited, although virus shedding was reduced. The gathered data from this study were restricted to viral characteristics, such as virus shedding, transmission frequency and morbidity and mortality due to the lack of ferret immunological reagents. From these data, an ideal state of protection against re-infection of influenza virus was defined along with a goal to generate a vaccine that will elicit similar protection. Although not sterilizing, it was found that seasonal A(H1N1) pre-immune animals exhibited immunity and mitigated infection against the pandemic A(H1N1) virus [70].

Through multiple infection and vaccination schemes, vaccination with the trivalent influenza vaccine (TIV; containing only one Type B strain instead of two) was unable to lessen the resulting morbidity or contact transmission in ferrets following challenge with the pandemic A(H1N1) [38]. Conversely, imprinting with a seasonal A(H1N1) virus altered the morbidity, but not the transmission characteristics of the pandemic A(H1N1) [38]. Although, these viral traits were muted, there was only minimal detection of cross-reactive serum antibodies.

The 2009 pandemic was characterized by distinct protective responses seen between different age groups of people. Older adults were more protected than young adults and children against the A(H1N1)/California/07/2009 pandemic virus. This older population was captured in the pre-immune ferret model by imprinting with historical antigenically distinct viruses [71]. The protective responses to a pandemic challenge were then measured. The historical viruses from the 1950s and earlier elicited more protective responses than the naïve ferrets [71]. This corroborated the human data that the older population was more protected than the younger to a pandemic challenge. Next, the effects of imprinting on the response to a pandemic vaccine were determined. Ferrets imprinted with seasonal A(H1N1) received a pandemic A(H1N1) vaccine. This seasonal A(H1N1) priming did not diminish the antibody response to either infection or vaccination with the pandemic virus [71]. Furthermore, original antigenic sin was not observed in the context of seasonal A(H1N1) to pandemic A(H1N1). Additionally, priming with seasonal A(H1N1) virus provided cross-protection against the pandemic virus. However, it did not impact the transmission efficiency [72].

A human’s pre-immune history can be recapitulated in ferrets by conducting repeated infections. Carter et al. [73] utilized this technique by sequentially infecting two different ferret groups with seasonal A(H1N1): one with historical A(H1N1) from 1934 to 1957, and another with contemporary A(H1N1) viruses from 1999 to 2007. The abilities of the differing pre-immunities to protect against an A(H1N1)/California/07/2009 challenge were compared. Both sequential groups were protected from challenge; they exhibited no weight loss, minimal recoverable virus, and no transmission compared to ferrets pre-immunized with only one of the viruses. Unique to sequentially infected ferrets, the elicited antibody profile was broader and interacted with pandemic A(H1N1) HA compared to the single pre-immunity groups as measured with HA-specific ELISA binding. The recall and adaptation of the antibody profile over time in response to sequential exposures is complex and still not completely understood. However, these interactions help to explain the puzzling epidemiological and serological observations surrounding the pandemic A(H1N1) outbreak. Carter et al. [73] hypothesized that the older adults were exposed to more antigenically variant strains and have extensive protection compared to young adults with less exposure.

In addition, the changes in the elicited antibody profile point to the possibility of achieving broad vaccine-induced protection against influenza viruses by sequential immunization with a series of antigenic variants. Further analysis with these ferret samples and infection/immunization scheme confirmed the change in antibody profile observed previously. Anti-HA stalk antibodies increased, even in the absence of receptor-binding site antibodies, leading to the observed cross-reactivity and reduction in clinical signs and transmission [74]. In contrast to previous reports that pre-immunity induced protection does not wane [68], the boosts in anti-HA stalk antibodies and the cross-reactivity induced from sequential infection with antigenically distinct seasonal A(H1N1) declined over time [74].

The pandemic outbreak occurred from a transmission event of an avian-human-swine reassortant virus from a swine host into the human population [75]. Whereas many groups investigated the effects of human seasonal A(H1N1) imprinting, the effects of imprinting with a classical swine virus were also determined. Min et al. [76] exhibited that infection with classical swine viruses elicited cross-reactive neutralizing antibody activity and provided protection against the pandemic A(H1N1) virus.

Over time, a natural break point occurred between the investigation into the pandemic mystery and the examination of the immune system response within the context of imprinting and pre-immunity. Within a seasonal and pandemic A(H1N1) sequential infection study, the changes in polyclonal serum antibodies responses were measured. Ferrets pre-immunized with the seasonal A(H1N1)/Texas/36/1991 were followed up with an A(H1N1)/California/07/2009 infection. The elicited serum antibody specificity shifted to target a different region of the HA compared to the A(H1N1)/Texas/36/1991 only serum [77]. The overall antibody response moved to epitopes near the HA receptor-binding domain; sites where homology between these two strains is shared [77]. These findings resulted in research in the basic science of how imprinting and pre-immunity affects vaccination and infection and subsequent immune responses.

### 7.2. Contemporary A(H1N1)/A(H3N2) Models

The current pre-immunity models are moving away from investigating the differences in disease symptoms and vaccine effectiveness observed in the human population in response to the pandemic virus. Resources are now focused on how pre-immunity effects vaccination responses and general vaccine efficacies to any virus, not just in terms of the pandemic A(H1N1) (Tables S2 and S3). Furthermore, the pre-immune model is now being used, in replacement of a naïve model, to test novel vaccine candidates and methods currently in research and development [37]. Sequential infections of antigenically distinct viruses lead to a broader antibody response than that of just one strain [59,78]. This illustrated the importance of using a pre-immune model for antigenic characterization and vaccine testing. In fact, when A(H3N2) antigenic maps were produced using sera from naïve or pre-immune ferrets the maps poorly correlated; the classification of whether two viruses were antigenically distinct or similar varied with the model (naïve or pre-immune) [79]. The broader antibody response characterized with A(H1N1) or A(H3N2) sequential infections was extremely informative at the hetero-subtype level [78]. However, the impacts of other subtypes, such as A(H5N1) and A(H7N9) were not investigated, and neither were the nuances of how protective different antigenically drifted strains within a subtype investigated.

The antibody profiles of sequentially infected ferrets with A(H3N2) viruses revealed that A(H3N2) pre-immunity affected both the quantity and quality of antibodies elicited [79]. With high HAI titers against the imprinting virus, lower titers were observed toward the most recent isolate, A(H3N2)/Hong Kong/4801/2014. After repeated A(H3N2) infections, the antibody avidities gradually increased for A(H3N2) compared to those from a single homologous infection. This repeated A(H3N2) exposure expanded the cross-reactivity breadth against the same HAI panel. With computationally optimized HA vaccines, the same increased breadth phenomenon, back-boosting, occurred. Pre-immunity to a historical A(H3N2) virus helped boost the magnitude and breadth of the broadly neutralizing antibodies elicited by computationally optimized broadly reactive antigen (COBRA) immunogens compared to a naïve ferret group [37]. Emphasis was placed on the concept that testing vaccine candidates in naïve ferrets do not reflect the performance of the vaccines in the human population. In an A(H1N1) primed model, greater protection after vaccination was observed [18].

Vaccine effectiveness varies in the human population from season to season. Epidemiological data from people suggests that pre-existing immunity can result in decreased vaccine effectiveness [80,81,82]. The pre-immune model has been used to study how vaccines can overcome pre-existing immunity to mount a new response with M2-deficient single replicon vaccine candidates for A(H1N1) and A(H3N2) subtypes [83]. Type B and A(H1N1) heterosubtypic pre-immunity followed by A(H3N2) vaccination provided protection against an antigenically distinct A(H3N2) challenge. A(H1N1) pandemic homosubtypic imprinting negatively affected the ability of a FluMist-like vaccine to elicit protection towards seasonal A(H1N1) [83]. These key findings highlight the difficulty with inducing immunity to a novel HA in the presence of pre-existing heterotypic, heterosubtypic, and homosubtypic immunity.

Although the questions surrounding the 2009 outbreak have been sufficiently answered, there still remains the possibility for a second swine-origin pandemic. Even of the same subtype and species origin, pandemic A(H1N1) pre-existing immunity was unable to induce sterilizing immunity to current circulating swine-origin A(H1N1) viruses [84]. Furthermore, other subtypes—A(H3N2) and A(H1N2)—circulate in swine and transmit into the human population [85]. The swine-origin A(H3N2) subtype raises concerns due to human’s documented susceptibility to A(H3N2) viruses, and transmission in ferrets being as efficient as human-origin seasonal A(H3N2) viruses [86]. Differing pre-immunity may be protective against these swine-origin viruses; pre-immunity with the human A(H3N2)/Perth/16/2009 cross-protected against a swine-origin variant A(H3N2), whereas other human strains did not [26]. Hence, prior seasonal virus infections may be protective by limiting viral replication and reducing transmission. This may suggest that within the human population, different age groups are more susceptible to certain transmission events depending on the subtypes of influenza virus that they have previously been exposed to [4].

The ferret immune response to reinfection is strikingly different compared to the response after a primary infection [87]. When comparing A(H1N1)/Mexico/4108/2009 challenge in naïve and A(H1N1)/Mexico/4108/2009 pre-immune ferrets, the pre-immunity status limited viral titers. The virus was still detectable at low levels at day seven post-infection. Assessment of the ferret transcriptome during this challenge provided invaluable data for unraveling the immune response to infection. In a primary challenge, innate immune system and inflammatory genes were upregulated in both the lung and lymph node tissues. Comparatively, in the pre-immune ferret, the adaptive immune response genes (CXCL10, CCL5) were upregulated in the lungs, with no upregulation in the lymph nodes. The lack of lymph node gene activity suggested that influenza specific CD8+ T-cells and B-cells may have originally resided within the lungs before infection or the adaptive immune response originated from another unidentified peripheral compartment [87].

Influenza virus infection also imprints on the influenza-specific T-cell memory compartment. In an A(H1N1)/A(H1N1) homologous and an A(H1N1)/A(H3N2) heterologous challenge, A(H1N1) imprinting partially protected against the A(H3N2) challenge, reducing virus shedding duration, but not the peak virus titer. The inflammatory immune response was increased, but less than that of the immunologically naive infected ferrets [88]. Differences between interferon-gamma (IFN-γ) producing peripheral blood mononuclear cells (PBMCs) in naïve vs. A(H1N1) pre-immunized ferrets with a homologous A(H1N1) challenge were not discernable. However, the pre-immune ferrets had IFN-γ producing PBMCs that were stimulated by heterosubtypic viruses. The imprinting event did not lessen the quantity IFN-γ producing PBMCs compared to mock/A(H3N2) infected ferrets. However, heterosubtypic pre-immunity affected the reactivity of the IFN-γ producing lung mononuclear cells (MNCs) and induced whole-blood IFN-γ producing cells quicker. A(H1N1)/A(H3N2) pre-immune ferrets had high reactivity of IFN-γ producing lung MNCs to A(H1N1) virus compared to A(H1N1)/A(H1N1) ferrets that had no increase. Although, the classification of these T-cells as CD8+ or CD4+ was not conducted as of yet, pre-immune ferrets can be used to model T-cell population contributions to influenza-specific memory and recognition.

Future studies will investigate the different cellular and humoral responses to influenza virus infection and clarify the differences between T-cell subsets. The study conducted by Hay et al. [89] analyzed data derived from a previous study [70] to mathematically model the short-term antibody kinetics from either influenza infection or vaccination with and without adjuvant. Their results, although limited in sample size, highlight the potential future applications and data analysis of the serological, cellular, and virological data that can be collected during a pre-immune study.

### 7.3. A(H5N1) Models

The effects of A(H1N1) and A(H3N2) pre-immunity on the A(H5N1) subtype or the effect of A(H5N1) on A(H1N1) or A(H3N2) vaccination or subsequent viral challenge are not well known. Although studies have been performed in pre-immune mice [90,91], few studies have addressed pre-immunity using the viruses of the A(H5N1) subtype [92] (Table S4). Pre-pandemic vaccines for A(H5N1) influenza viruses elicited low seroconversion proportions in immunologically naïve ferrets. When primed with seasonal live attenuated influenza vaccine (LAIV), A(H5N1) HA-specific IgG antibody secreting cells (ASC) were stimulated compared to unprimed ferrets. Expansion of the A(H1N1) or A(H3N2) specific memory B-cells may cross-react with the A(H5N1) HA antigen. When imprinted with the individual vaccine strains, the IgG ASC levels were similar to the LAIV imprinted cells, with the A(H1N1) imprinting influenza virus eliciting higher responses than A(H3N2) imprinting viruses. This effect may be a response to the HA molecule rather than the A(H1N1) NA protein because an A(H1N2) reassortant virus elicited a similar response. But a synergistic or additive effect was not determined with an A(HXN1) reassortant virus to confirm the lack of protective contribution from the NA. However, upon closer inspection, the protection afforded by the A(H1N1) HA was temporary and waned to levels similar to naïve ferrets three weeks after the second dose was administered.

## 8. Other Pre-Immune Influenza Animal Models

Other animal models are used to study influenza vaccination and infection. The mouse model is commonly used due to the plethora of genetic tools and the availability of reagents. In addition, pre-immunity can be established in the mouse model [23,76,78,91,93,94,95,96]. Within mice, pre-infection compared to vaccination elicited similar innate immunity and antibody responses. Sterilizing immunity was achieved in pre-immune animals that had reduced viral receptors and increased T-cell responses in the lungs [41]. Although less utilized than mice or ferrets, guinea pigs produce similar results [78,97].

## 9. Design Considerations for Pre-Immune Ferret Studies

### 9.1. Priming and Pre-Immune Strain Selection

The main hypothesis and question being addressed will determine strains selected for use in a pre-immune model. The priming method, whether by vaccination or infection should be evaluated based on the target population of the vaccine/study. An infant or child target demographic may warrant that priming actually occur through vaccination followed by viral challenge. This instance recapitulates if the vaccine was administered before exposure to influenza. In contrast, to target populations whose first exposure is through infection, live influenza virus would be the appropriate priming method.

The initial imprinting strain should be antigenically representative for the population being modeled. Therefore, the timing and order of the infections are variable. After imprinting, ferrets may be re-infected to add to the pre-immune history or be vaccinated or challenged according to study design. However, the magnitude of the contributions of a full pre-immunity history compared to just the initial imprinting virus on the immune response has not been adequately quantified. For instance, if attempting to recapitulate a person born in 1970, it is unclear if it is only necessary to pre-immunize with an A(H3N2) 1970s virus, followed by A(H1N1) influenza virus, or if all of the antigenically distinct A(H3N2) viruses are needed to establish a true pre-immune state. Studies investigating the effects of heterosubtypic and heterotypic imprinting and pre-immunity can be expanded to include: (1) Type B imprinting followed by an A(H1N1) or A(H3N2) vaccination or challenge, (2) A(H2N2) imprinting, (3) A(H1N1) imprinting and A(H3N2) pre-immunity and vice versa on which combination results in more protection against A(H1N1), A(H3N2), A(H2N2), or A(H5N1) challenge. Limited research is available on the serological, cellular, and immunological effects of infections with varying infection doses, but there have been observed differences [98].

### 9.2. Immune System Cool-Down

The immunological system should return to the baseline immunological state before attempting another repeat infection or vaccination. This period should encompass the cool-down time for both the innate and adaptive immune systems, including the induction and contraction of B- and T-cells into their memory states. Studies similar to Leon et al. [87] are of great importance for understanding the inner workings of the ferret immune system during imprinting and re-infection. Without these studies, this cool-down component will need to continuously be stated as a study limitation. For example, Pulit-Penaloza et al. [84] indicated that a thirty-one day interval between primary and secondary challenges for their studies may have been too short, allowing for elevated non-specific innate responses from the primary infection to affect the secondary. Without letting the immune system return to baseline, misrepresentation of cross-protection may be observed. Cheng et al. [92] found that the heterologous protective titers waned to levels not significantly different than the negative naïve control ferret group providing only temporary protection.

### 9.3. Age/Gender/Vendor Specific Responses

The age and gender of the animals being used may be a confounding factor especially when comparing humoral responses [99,100]. The ages for male and female ferrets ranged from two to twelve months. Ferrets were considered aged when they were greater than four years of age [36]. Reporting results by gender may reveal new avenues for influenza virus research. The breeding vendor and housing conditions of the animals should also be reported. Within the mouse model, the microbiome differs by vendor in the gut [101,102] and in the lungs [103], contributing to different responses to vaccination and influenza infection [104,105,106]. Therefore, it is recommended that when mice or ferrets are housed in separate holding rooms, bedding or enrichment equipment is shared between the cages to merge the microbiomes together [105]. Inclusion of the ferret health history is also beneficial. Metadata such as whether they were castrated, spayed, de-scented, or received any previous vaccinations or treatments may shed light on immunological results.

The ferret model captures special at-risk populations. Ferret age was varied to encompass different age groups: young, adult and old. Adult and aged ferrets, similar to humans, exhibit significant immune response differences when comparing homologous and heterologous A(H1N1) priming and challenges suggesting immune senescence in the aged ferret population [36]. Therefore, the use of this model would contribute to vaccine testing and efficacy studies. Currently, a vaccine specifically formulated for aged individuals already exists due to their high-risk status and substantial contributions to influenza-associated hospitalizations and deaths [33,107,108]; this high dose vaccine contains 60 ug of each vaccine strain HA, compared to the standard 15 ug. The National Institute of Allergy and Infectious Diseases’ (NIAID) goal for the development of a universal influenza vaccine requires a protection equal to or greater than 75% against symptomatic disease lasting at least one year in all populations, including, at-risk populations [109]. The addition of pre-existing immune responses to these various at-risk population models more accurately reflects the variation of the human population and allows for appropriate testing of novel vaccine candidates.

## 10. Pre-Immunity on the Immune Response

The effects of imprinting and pre-immunity on subsequent humoral and cellular responses are still under investigation. During initial pre-immune ferret infection, there are increased nasal protein secretions compared to naïve ferrets during a heterologous challenge [39]. Characterization of the influenza virus infectome during different stages of the infectious process, with and without prior specific immunity to influenza, has recently been reported [87]. The differences in protection of a pre-immune animal compared to a naïve animal may be due to recall of antibodies specific to shared epitopes which do not necessary need to be neutralizing [110]. Infection induces T-cell responses to T-cell epitopes within the HA and other proteins, and even neuraminidase inhibiting antibodies that are not elicited by split-inactivated vaccines [41,111]. These responses may be either synergistic or antagonistic when paired with vaccination. For instance, vaccination may boost the non-neutralizing antibodies leading to decreased vaccine efficacy compared to a naïve animal.

This back-boosting, also described as an anamnestic response, has been observed in the human population [112,113,114,115] and has been recapitulated within the ferret model. This similarity makes it a useful tool for determining vaccine performance in a setting where back-boosting is present [37]. Specifically, back-boosting was observed when imprinted with A(H1N1)/Singapore/6/1986 followed by A(H1N1)/California/07/2009 VLP vaccination. The breadth of HAI-specific antibody response was wider than just A(H1N1)/Singapore/6/1986 alone and covered more viruses. The breadth increased to include viruses before and after the 1986 seasonal virus [59]. The back-boosting observed after a VLP-vaccination was dependent on the recognition of memory B-cell and T-helper cell epitopes specific to the HA of the virus. This increase in breadth was also observed after sequential infections with ELISA titers to total antibody binding [78]. This back-boosting is what contributed to the difference in the naïve vs. pre-immune antigenic maps of different A(H3N2) viruses [79]. The exact mechanism of back-boosting is not completely elucidated [113].

Heterosubtypic protection between the Type A influenza strains suggest that cross-reactive cellular immune responses may be contributing to virus control [83]. Protective T-cell responses are elicited through recognition of cross-reactive epitopes [84,88]. Cross-reactive memory B-cells can also be elicited post-imprinting [63]. Characterization of the cellular and humoral responses, similar to the human-based study conducted by Ryan et al. [116], with imprinted and sequentially infected ferrets is a priority to determine if cellular immune responses differ with A(H1N1) and A(H3N2) subtype infections [116], or during A(H3N2) and A(H5N1) influenza virus co-infections [117], or with seasonal vs. pandemic A(H1N1) influenza virus infections [118,119].

As the reagents for the ferret animal model continues to expand, the opportunities to investigate different correlates of infection and/or protection magnify. Before this reagent development, research was limited to characterizing serum and nasal washes for neutralizing antibodies and measuring clinical signs after infection. Therefore, future studies can capture the cellular immune reactions of T-cell responses with IFN-γ ELISAs [120], peripheral blood leukocyte tracking [121], and ELISpots [88,122], as well as the humoral immunity [18,92,123,124].

## 11. Validation and Further Work of the Pre-Immune Model

The pre-immune ferret model is within its early stages of development; to become a mainstay within the scientific community and used reliably in the context of influenza virus research, the baseline effects of infection, re-infection, and vaccination within the ferret need to be addressed first. This involves determining the cool-down time of the ferret immune system following infection and the differences compared to a vaccination. Furthermore, studies of the different immunological ferret responses such as cytokine quantity and diversity, B- and T-cell repertoire, and B- and T-cell recall responses are important to characterize. In addition, advancements in high-throughput, single cell sequencing technology allow depiction of the B-cell evolution from a single ancestral B-cell [124].

The ferret model is used to study influenza virus infection because of the ferret’s natural susceptibility, shared clinical signs of illness, and possession of similarities in respiratory physiology, cell composition, and distribution of sialic acid receptors. Although present, these components may not interact in the same manner as human immune system following influenza virus infection [78]. Comparisons of different pre-immune influenza animal models (mice, guinea pigs, and ferrets) to human serology data emphasized that the use of different animal models should be heavily considered for pre-clinical vaccine studies due to the biases between them. Basic research of the ferret physiology can be compared and validated against data from human studies. Cross-validation will either bolster the ferret findings or they will provide researchers the ability to determine which immunological findings are relevant for further investigation and which are solely ferret-specific phenomena. This process focuses on the ferret model being a surrogate for the human. The decision-making process of vaccine selection and therapeutics relies on the ferret, and it is important that human-ferret shared traits are appropriately distinguished. Therefore, further study, comparison, and validation are needed to fully grasp the predispositions and limitations of the model.

## 12. Conclusions

Although initially developed in the 1970s, recently, the pre-immune ferret model has rapidly progressed into a vital tool for the development of a broadly neutralizing influenza vaccine. In conclusion, care needs to be taken to begin planning studies to incorporate the effects of imprinting and pre-immunity within the animal model to apply the results to the human system. Animal models used for influenza research are available and widely use. However, as more findings and reagents become available, the models need to be updated appropriately. In addition, more research on the immunological effects of imprinting in ferrets, which is then compared to the research on the effects of imprinting on humans, will contribute to the validation of using the ferret as an appropriate animal model to study influenza virus in humans. Altogether, the major goal of developing a broadly neutralizing influenza vaccine by priming the immune system adequately to produce the same sterilizing immunity as infection, but without the deleterious effects, could be tested in these models.

## Supplementary Materials

The following are available online at https://www.mdpi.com/2076-393X/8/2/173/s1, Table S1. Pre-immune ferret studies with only A(H1N1) and Type B influenza in published peer-reviewed literature. Legend: Underlined = Vaccination; Bold = Live infection; TIV = Trivalent inactivated vaccine; LAIV = Live attenuated influenza vaccine; IIV = Inactivated influenza vaccine; VLP = virus-like particle, Table S2. Pre-immune ferret studies with A(H1N1), A(H3N2), and Type B influenza in published peer-reviewed literature. Legend: Underlined = Vaccination; Bold = Live infection; TIV = Trivalent inactivated vaccine; LAIV = Live attenuated influenza vaccine; IIV = Inactivated influenza vaccine; VLP = virus-like particle, Table S3. Pre-immune ferret studies with only A(H3N2) and Type B influenza in published peer-reviewed literature. Legend: Underlined = Vaccination; Bold = Live infection; TIV = Trivalent inactivated vaccine; LAIV = Live attenuated influenza vaccine; IIV = Inactivated influenza vaccine; VLP = virus-like particle, Table S4. Pre-immune ferret studies with A(H5N1) influenza in published peer-reviewed literature. Legend: Underlined = Vaccination; Bold = Live infection; TIV = Trivalent inactivated vaccine; LAIV = Live attenuated influenza vaccine; IIV = Inactivated influenza vaccine; VLP = virus-like particle.
