# Supplementary material for: Immune Imprinting in the Influenza Ferret Model
Source: Vaccines (Basel). 2020 Apr 8;8(2):173. doi: 10.3390/vaccines8020173 (PMC7348859; doi:10.3390/vaccines8020173)
Supplement: Supplementary file 1 [file vaccines-08-00173-s001.pdf]

vaccines

MDPI

Review

Immune Imprinting in the Influenza Ferret Model

Amanda Lee Skarlupka [1] and Ted Ross [1,2,*]

Table 1. Pre-immune ferret studies with only A(H1N1) and Type B influenza in published peer-reviewed literature. Legend: Underlined = Vaccination; Bold = Live infection; TIV = Trivalent inactivated vaccine; LAIV = Live attenuated influenza vaccine; IIV = Inactivated influenza vaccine; VLP = virus-like particle.

Study	Ferret			Exposures							Samples Taken	Assays conducted
	Sex	Age	Source	1st	Time	2nd	Time	3rd	Time	4th		
Min, J. Y., et al. (2010) [76]	Unknown	8-12 week	Unknown	**A(H1N1)/California/04/2009**	28 day	**A(H1N1)/California/07/2009**					Nasal turbinate, lungs, brain tissue	Virus titer; Hemagglutinin Inhibition; Neutralization
				**A(H1N1)/California/07/2009**	28 day	**A(H1N1)/California/07/2009**						
				**A(H1N1)/New Jersey/8/1976**	28 day	**A(H1N1)/California/07/2009**						
				**A(H1N1)/swine/Iowa/1931**	28 day	**A(H1N1)/California/07/2009**						
O'Donnell, C. D., et al. (2012) [71]	Male and Female	8-12 week	Triple F Farms	**A(H1N1)/Alaska/1935**	6 week	**A(H1N1)/California/07/2009**					Nasal wash; Serum; Blood - PBMCs; Spleens - Splenocytes	Hemagglutinin Inhibition; Neutralization; Pseudovirion luciferase assay; HA:NA-specific IgG and IgM ELISA
				**A(H1N1)/Fort Monmouth/1/1947**	6 week	**A(H1N1)/California/07/2009**						
				**A(H1N1)/Fort Warren/1/1950**	6 week	**A(H1N1)/California/07/2009**						
				**A(H1N1)/New Jersey/8/1976**	6 week	**A(H1N1)/California/07/2009**						
				**A(H1N1)/New Caledonia/20/1999**	6 week	**A(H1N1)/California/07/2009**						
				**A(H1N1)/California/07/2009**	6 week	**A(H1N1)/California/07/2009**						
				**B(Vic)/Malaysia/2504/2004**	6 week	**A(H1N1)/California/07/2009**						

Leon, A. J., et al. (2013) [87]	Male	Unknown	In-house ferret breeding	**A(H1N1)/Mexico/4108/2009**	21 day	**A(H1N1)/Mexico/4108/2009**					Lungs; Lymph nodes; RNA	mRNA sequencing; Digital gene expression; Real-time PCR
Kirchenbaum, G. A., et al. (2016) [74]	Female	6-12 month	Marshall Farms or Triple F Farms	**A(H1N1)/Puerto Rico/8/1934**	84 day	**A(H1N1)/Puerto Rico/8/1934**	84 day	**A(H1N1)/California/07/2009**			Nasal wash; Serum; Blood - PBMC; Paratracheal lymph nodes	Virus titer; Hemagglutinin Inhibition; HA-specific ELISA; HA-specific Competition ELISA
				**A(H1N1)/Puerto Rico/8/1934**	84 day	**A(H1N1)/Denver/1/1957**	84 day	**A(H1N1)/California/07/2009**				
				**A(H1N1)/Puerto Rico/8/1934**	84 day	**A(H1N1)/Brisbane/59/2007**	84 day	**A(H1N1)/California/07/2009**				
				**A(H1N1)/Singapore/6/1986**	84 day	**A(H1N1)/New Caledonia/20/1999**	84 day	**A(H1N1)/California/07/2009**				
				**A(H1N1)/Singapore/6/1986**	84 day	**A(H1N1)/Brisbane/59/2007**	84 day	**A(H1N1)/California/07/2009**				
				**A(H1N1)/Puerto Rico/8/1934**	84 day	**A(H1N1)/Fort Monmouth/1/1947**	84 day	**A(H1N1)/Denver/1/1957**	84 day	**A(H1N1)/California/07/2009**		
				**A(H1N1)/Texas/36/1991**	84 day	**A(H1N1)/New Caledonia/20/1999**	84 day	**A(H1N1)/Brisbane/59/2007**	84 day	**A(H1N1)/California/07/2009**		
O'Donnell, C.D., et al. (2014) [71]	Male and Female	8-12 week	Triple F Farms	**A(H1N1)/Alaska/1935**	6 week	**A(H1N1)/California/07/2009**					Serum; PBMCs	Hemagglutinin Inhibition; Neutralization; HA:NA-specific IgG ELISA; IFN-gamma ELISpot
				**A(H1N1)/Fort Monmouth/1/1947**	6 week	**A(H1N1)/California/07/2009**						
				**A(H1N1)/Fort Warren/1/1950**	6 week	**A(H1N1)/California/07/2009**						
				**A(H1N1)/New Jersey/8/1976**	6 week	**A(H1N1)/California/07/2009**						
				**A(H1N1)/New Caledonia/20/1999**	6 week	**A(H1N1)/California/07/2009**						
				**A(H1N1)/California/07/2009**	6 week	**A(H1N1)/California/07/2009**						
				**B(Vic)/Malaysia/2504/2004**	6 week	**A(H1N1)/California/07/2009**						
				**A(H1N1)/Alaska/1935**	6 week	A(H1N1)/California/07/2009 LIAV						

				**A(H1N1)/Fort Monmouth/1/1947**	6 week	A(H1N1)/California/07/2009 LIAV						
				**A(H1N1)/Fort Warren/1/1950**	6 week	A(H1N1)/California/07/2009 LIAV						
				**A(H1N1)/New Jersey/8/1976**	6 week	A(H1N1)/California/07/2009 LIAV						
				**A(H1N1)/New Caledonia/20/1999**	6 week	A(H1N1)/California/07/2009 LIAV						
				**A(H1N1)/California/07/2009**	6 week	A(H1N1)/California/07/2009 LIAV						
				**B(Vic)/Malaysia/2504/2004**	6 week	A(H1N1)/California/07/2009 LIAV						
				**A(H1N1)/Alaska/1935**	6 week	A(H1N1)/California/07/2009 IIV + Adjuvant						
				**A(H1N1)/Fort Monmouth/1/1947**	6 week	A(H1N1)/California/07/2009 IIV + Adjuvant						
				**A(H1N1)/Fort Warren/1/1950**	6 week	A(H1N1)/California/07/2009 IIV + Adjuvant						
				**A(H1N1)/New Jersey/8/1976**	6 week	A(H1N1)/California/07/2009 IIV + Adjuvant						
				**A(H1N1)/New Caledonia/20/1999**	6 week	A(H1N1)/California/07/2009 IIV + Adjuvant						
				**A(H1N1)/California/07/2009**	6 week	A(H1N1)/California/07/2009 IIV + Adjuvant						
				**B(Vic)/Malaysia/2504/2004**	6 week	A(H1N1)/California/07/2009 IIV + Adjuvant						
Carter, D. M., et al. (2017) [59]	Female	6-12 month	Triple F Farms	**A(H1N1)/Singapore/6/1986**	84 day	**A(H1N1)/New Caledonia/20/1999**	84 day	P1-COBRA HA VLP + Adjuvant			Nasal wash; Serum	Virus Titer; Hemagglutinin Inhibition; Neutralization; H1 HA stem-
				**A(H1N1)/Singapore/6/1986**	84 day	**A(H1N1)/New Caledonia/20/1999**	84 day	X3-COBRA VLP + Adjuvant				

				**A(H1N1)/Singapore/6/1986**	84 day	**A(H1N1)/New Caledonia/20/1999**	84 day	A(H1N1)/California/07/2009 VLP + Adjuvant				specific IgG ELISA
				**A(H1N1)/Singapore/6/1986**	84 day	**A(H1N1)/Brisbane/59/2007**	84 day	P1-COBRA VLP + Adjuvant				
				**A(H1N1)/Singapore/6/1986**	84 day	**A(H1N1)/Brisbane/59/2007**	84 day	X3-COBRA VLP + Adjuvant				
				**A(H1N1)/Singapore/6/1986**	84 day	**A(H1N1)/Brisbane/59/2007**	84 day	A(H1N1)/California/07/2009 VLP + Adjuvant				
				**A(H1N1)/Singapore/6/1986**	84 day	P1-COBRA HA VLP + Adjuvant	84 day	P1-COBRA HA VLP + Adjuvant				
				**A(H1N1)/Singapore/6/1986**	84 day	X3-COBRA VLP + Adjuvant	84 day	X3-COBRA VLP + Adjuvant				
				**A(H1N1)/Singapore/6/1986**	84 day	A(H1N1)/California/07/2009 VLP + Adjuvant	84 day	A(H1N1)/California/07/2009 VLP + Adjuvant				
				**A(H1N1)/California/07/2009**	84 day	P1-COBRA HA VLP + Adjuvant	84 day	P1-COBRA HA VLP + Adjuvant				
				**A(H1N1)/California/07/2009**	84 day	X3-COBRA VLP + Adjuvant	84 day	X3-COBRA VLP + Adjuvant				
				**A(H1N1)/California/07/2009**	84 day	A(H1N1)/California/07/2009 VLP + Adjuvant	84 day	A(H1N1)/California/07/2009 VLP + Adjuvant				
				**A(H1N1)/Brisbane/59/2007**	84 day	P1-COBRA HA VLP + Adjuvant	84 day	P1-COBRA HA VLP + Adjuvant				
				**A(H1N1)/Brisbane/59/2007**	84 day	X3-COBRA VLP + Adjuvant	84 day	X3-COBRA VLP + Adjuvant				
				**A(H1N1)/Brisbane/59/2007**	84 day	A(H1N1)/California/07/2009 VLP + Adjuvant	84 day	A(H1N1)/California/07/2009 VLP + Adjuvant				
				**P1-COBRA HA:A(H1N1)/California/07/2009 NA: A(H1N1)/Puerto Rico/8/1934**	84 day	P1-COBRA HA VLP + Adjuvant	84 day	P1-COBRA HA VLP + Adjuvant				

				**P1-COBRA HA:A(H1N1)/California/07/2009 NA: A(H1N1)/Puerto Rico/8/1934**	84 day	X3-COBRA VLP + Adjuvant	84 day	X3-COBRA VLP + Adjuvant				
				**P1-COBRA HA:A(H1N1)/California/07/2009 NA: A(H1N1)/Puerto Rico/8/1934**	84 day	A(H1N1)/California/07/2009 VLP + Adjuvant	84 day	A(H1N1)/California/07/2009 VLP + Adjuvant				
				**X3-COBRA HA:A(H1N1)/California/07/2009 NA: A(H1N1)/Puerto Rico/8/1934**	84 day	P1-COBRA HA VLP + Adjuvant	84 day	P1-COBRA HA VLP + Adjuvant				
				**X3-COBRA HA:A(H1N1)/California/07/2009 NA: A(H1N1)/Puerto Rico/8/1934**	84 day	X3-COBRA VLP + Adjuvant	84 day	X3-COBRA VLP + Adjuvant				
				**X3-COBRA HA:A(H1N1)/California/07/2009 NA: A(H1N1)/Puerto Rico/8/1934**	84 day	A(H1N1)/California/07/2009 VLP + Adjuvant	84 day	A(H1N1)/California/07/2009 VLP + Adjuvant				
				**A(H1N1)/Singapore/6/1986**	168 day	**A(H1N1)/California/07/2009**		-				
				**A(H1N1)/Singapore/6/1986**	84 day	P1-COBRA HA VLP + Adjuvant	84 day	**A(H1N1)/California/07/2009**				
				**A(H1N1)/Singapore/6/1986**	84 day	X3-COBRA VLP + Adjuvant	84 day	**A(H1N1)/California/07/2009**				
				**A(H1N1)/Singapore/6/1986**	84 day	A(H1N1)/California/07/2009 VLP + Adjuvant	84 day	**A(H1N1)/California/07/2009**				
Li, Y., et al. (2013) [77]	Female	6-12 month	Marshall Farms	**A(H1N1)/Fort Monmouth/1/1947**	84 day	**A(H1N1)/California/07/2009**					Serum	Hemagglutinin Inhibition

				**A(H1N1)/Denver/1/1957**	84 day	**A(H1N1)/California/07/2009**						
				**A(H1N1)/Texas/36/1991**	84 day	**A(H1N1)/California/07/2009**						
				**A(H1N1)/Brisbane/59/2007**	84 day	**A(H1N1)/California/07/2009**						
Paquette, S. G., et al. (2014) [36]	Male	Adult: 4-6 month; Aged: >4 years	UHN Toronto, ON, Canada	**A(H1N1)/Mexico/4108/2009**	46 day	**A(H1N1)/California/07/2009**					Nasal Wash; PBMCs; RNA	Virus titer; Hemagglutinin inhibition; quantitative real-time PCR
				**A(H1N1)/Brisbane/59/2007**	39 day	**A(H1N1)/Mexico/4108/2009**						
Pulit-Penaloza, J. A., et al. (2018) [84]	Male	5-7 month	Triple F Farms	**A(H1N1)/California/07/2009 Respiratory droplet transmission**	31 day	**A(H1N1)/California/07/2009**					Nasal wash; trachea; lungs; Serum	Virus titer; Hemagglutinin Inhibition
				**A(H1N1)/California/07/2009 Respiratory droplet transmission**	31 day	**A(H1N1)/Ohio/09/2015**						

Table 2. Pre-immune ferret studies with A(H1N1), A(H3N2), and Type B influenza in published peer-reviewed literature. Legend: Underlined = Vaccination; Bold = Live infection; TIV = Trivalent inactivated vaccine; LAIV = Live attenuated influenza vaccine; IIV = Inactivated influenza vaccine; VLP = virus-like particle.

**Study**	**Ferret**			**Influenza Exposures**									**Samples Taken**	**Assays conducted**
	**Sex**	**Age**	**Source**	**1st**	**Time**	**2nd**	**Time**	**3rd**	**Time**	**4th**	**Time**	**5th**		
McLaren, C. and C. W. Potter (1974) [39]	Unknown	Young	Wellcome Research Laboratories, Beckenham: Immunized against canine distember virus a few weeks before the beginning of experiment	**A(H1N1)/Puerto Rico/8/1934**	7 week	A(H3N2)/Hong Kong/X31/1968 IIV	5 week	**A(H3N2)/Hong Kong/X31/1968**					Nasal wash; Serum	Virus titer; nasal wash protein estimate; Hemagglutinin Inhibition; Neutralization
				**B/Ann Arbor/1966**	5 week	A(H3N2)/Hong Kong/X31/1968 IIV	5 week	**A(H3N2)/Hong Kong/X31/1968**						
				A(H3N2)/Hong Kong/X31/1968 IIV	5 week	**A(H3N2)/Hong Kong/X31/1968**								

Yetter, R. A., et al. (1980) [68]	Male	adult	Marshal Research Animals, Inc. North Rose N.Y	**A(H3N2)/Port Chalmers/1973**	21 day	**A(H3N2)/Port Chalmers/1973**							Nasal Wash; Serum	Virus titer; Hemagglutin in Inhibition
					21 day	**A(H1N1)/Puerto Rico/8/1934**								
					18 month	**B/Lee/1940**								
				**A(H1N1)/Puerto Rico/8/1934**	21 day	**A(H3N2)/Port Chalmers/1973**								
					21 day	**A(H1N1)/Puerto Rico/8/1934**								
					18 month	**A(H3N2)/Port Chalmers/1973**								
Laurie, K. L., et al. (2010) [70]	Male and Female	500-1500g	Independent Breeders	A(H1N1)/Brisbane/59/2007-like; A(H3N2)/Uruguay/716/2007; B(Yam)/Florida/4/2006-like TIV (2008/2009 Northern Hemisphere) + Adjuvant	2 week	A(H1N1)/Brisbane/59/2007-like; A(H3N2)/Uruguay/716/2007; B(Yam)/Florida/4/2006-like TIV (2008/2009 Northern Hemisphere) + Adjuvant	5 week	**A(H1N1)/Fukushima/141/2006**					Nasal wash; Serum	Virus titer; Hemagglutin in Inhibition; Neutralization

				A(H1N1)/Brisbane/59/2007-like; A(H3N2)/Uruguay/716/2007; B(Yam)/Florida/4/2006-like TIV (2008/2009 Northern Hemisphere) + Adjuvant	2 week	A(H1N1)/Brisbane/59/2007-like; A(H3N2)/Uruguay/716/2007; B(Yam)/Florida/4/2006-like TIV (2008/2009 Northern Hemisphere) + Adjuvant	5 week	**A(H1N1)/California/07/2009**	10 week	**A(H1N1)/California/07/2009**				
				A(H1N1)/California/07/2009 IIV + Adjuvant	2 week	A(H1N1)/California/07/2009 IIV + Adjuvant	5 week	**A(H1N1)/Fukushima/141/2006**						
				A(H1N1)/California/07/2009 IIV + Adjuvant	2 week	A(H1N1)/California/07/2009 IIV + Adjuvant	5 week	**A(H1N1)/California/07/2009**						
				**A(H1N1)/Fukushima/141/2006**	8 week	**A(H1N1)/California/07/2009**								
				**A(H1N1)/Fukushima/141/2006**	8 week	**A(H3N2)/Panama/2007/1999**								
				**A(H1N1)/Fukushima/141/2006**	8 week	**A(H1N1)/Fukushima/141/2006**								
				**A(H1N1)/Auckland/1/2009**	8 week	**A(H1N1)/Fukushima/141/2006**								
				**A(H1N1)/Auckland/1/2009**	8 week	**A(H1N1)/California/07/2009**								
				**A(H1N1)/Auckland/1/2009**	8 week	**A(H3N2)/Panama/2007/1999**								

				**A(H3N2)/Panama/2007/1999**	8 week	**A(H1N1)/Fukushima/141/2006**								
				**A(H3N2)/Panama/2007/1999**	8 week	**A(H1N1)/California/07/2009**								
				**A(H3N2)/Panama/2007/1999**	8 week	**A(H3N2)/Panama/2007/1999**								
				**A(H1N1)/Fukushima/141/2006**	8 week	**A(H3N2)/Panama/2007/1999**	8 week	**A(H1N1)/Auckland/1/2009**						
				**A(H3N2)/Panama/2007/1999**	8 week	**A(H1N1)/Fukushima/141/2006**	8 week	**A(H1N1)/Auckland/1/2009**						
				**A(H3N2)/Panama/2007/1999**	4 week	A(H1N1)/Brisbane/59/2007-like; A(H3N2)/Uruguay/716/2007; B(Yam)/Florida/4/2006-like TIV (2008/2009 Northern Hemisphere) + Adjuvant	2 week	A(H1N1)/Brisbane/59/2007-like; A(H3N2)/Uruguay/716/2007; B(Yam)/Florida/4/2006-like TIV (2008/2009 Northern Hemisphere) + Adjuvant	2 week	**A(H1N1)/Fukushima/141/2006**	8 week	**A(H1N1)/Auckland/1/2009**		

Pearce, M. B., et al. (2011) [72]	Male	7-12 month	Triple F Farms	A(H1N1)/South Dakota/6/2007; A(H3N2/Uruguay/716/2007; B(Yam)/Florida/4/2006 LAIV (2008/2009 Northern Hemisphere)	4 week	A(H1N1)/South Dakota/6/2007; A(H3N2/Uruguay/716/2007; B(Yam)/Florida/4/2006 LAIV (2008/2009 Northern Hemisphere)	4.5 week	**A(H1N1)/South Dakota/6/2007**					Nasal wash; Serum	Virus titer; Hemagglutinin Inhibition; Neutralization
				A(H1N1)/South Dakota/6/2007; A(H3N2/Uruguay/716/2007; B(Yam)/Florida/4/2006 LAIV (2008/2009 Northern Hemisphere)	4 week	A(H1N1)/South Dakota/6/2007; A(H3N2/Uruguay/716/2007; B(Yam)/Florida/4/2006 LAIV (2008/2009 Northern Hemisphere)	4.5 week	**A(H1N1)/Mexico/4482/2009**						
				**A(H1N1)/Brisbane/59/2007**	8.5 week	**A(H1N1)/South Dakota/6/2007**								
				**A(H1N1)/Brisbane/59/2007**	8.5 week	**A(H1N1)/Mexico/4482/2009**								

Ellebedy, A. H., et al. (2011) [38]	Male and Female	3-4 month	Triple F Farms	A(H1N1)/Brisbane/59/2007-like; A(H3N2)/Uruguay/716/2007; B(Yam)/Florida/4/2006-like TIV (2008/2009 Northern Hemisphere)	3 week	A(H1N1)/Brisbane/59/2007-like; A(H3N2)/Uruguay/716/2007; B(Yam)/Florida/4/2006-like TIV (2008/2009 Northern Hemisphere)	3 week	**A(H1N1)/Tennessee/1-560/2009**					Nasal wash; Serum	Virus titer; Hemagglutinin Inhibition; Neutralization; Virus-specific IgG ELISA
				A(H1N1)/Brisbane/59/2007-like; A(H3N2)/Uruguay/716/2007; B(Yam)/Florida/4/2006-like TIV (2008/2009 Northern Hemisphere)	3 week	A(H1N1)/Brisbane/59/2007-like; A(H3N2)/Uruguay/716/2007; B(Yam)/Florida/4/2006-like TIV (2008/2009 Northern Hemisphere)	3 week	**A(H1N1)/Brisbane/59/2007**						
				**A(H1N1)/Brisbane/59/2007**	3 week	A(H1N1)/Brisbane/59/2007-like; A(H3N2)/Uruguay/716/2007; B(Yam)/Florida/4/2006-like TIV (2008/2009 Northern Hemisphere)	3 week	**A(H1N1)/Tennessee/1-560/2009**						

				A(H1N1)/Brisbane/59/2007-like; A(H3N2)/Uruguay/716/2007; B(Yam)/Florida/4/2006-like TIV (2008/2009 Northern Hemisphere)	3 week	**A(H1N1)/Tennessee/1-560/2009**								
Carter, D. M., et al. (2013) [73]	Female	6-12 month	Marshall Farms	**A(H1N1)/Puerto Rico/8/1934**	3 month	**A(H1N1)/California/07/2009**							Nasal wash; Serum	Virus titer; Hemagluttinin Inhibition; Neutralization; HA-specific IgG ELISA; Surface plasmon resonance;
				**A(H1N1)/Fort Monmouth/1/1947**	3 month	**A(H1N1)/California/07/2009**								
				**A(H1N1)/Denver/1/1957**	3 month	**A(H1N1)/California/07/2009**								
				**A(H1N1)/Texas/36/1991**	3 month	**A(H1N1)/California/07/2009**								
				**A(H1N1)/New Caledonia/20/1999**	3 month	**A(H1N1)/California/07/2009**								
				**A(H1N1)/Brisbane/59/2007**	3 month	**A(H1N1)/California/07/2009**								
				**A(H1N1)/Puerto Rico/8/1934**	3 month	**A(H1N1)/Fort Monmouth/1/1947**	3 month	**A(H1N1)/Denver/1/1957**	1 month	**A(H1N1)/California/07/2009**				
				**A(H1N1)/Texas/36/1991**	3 month	**A(H1N1)/New Caledonia/20/1999**	3 month	**A(H1N1)/Brisbane/59/2007**	1 month	**A(H1N1)/California/07/2009**				

				**A(H1N1)/Puerto Rico/8/1934**	3 month	**A(H1N1)/Puerto Rico/8/1934**	3 month	**A(H1N1)/Puerto Rico/8/1934**	1 month	**A(H1N1)/California/07/2009**				
				**A(H1N1)/Fort Monmouth/1/1947**	3 month	**A(H1N1)/Fort Monmouth/1/1947**	3 month	**A(H1N1)/Fort Monmouth/1/1947**	1 month	**A(H1N1)/California/07/2009**				
Nachbagauer, R., et al. (2017) [78]	Unknown	Unknown	Unknown	**A(H1N1)/New Caledonia/20/1999**	6 week	**A(H1N1)/California/04/2009**							Serum	HA- and NA-specific IgG ELISA
				**A(H3N2)/Philippines/2/1982**	6 week	**A(H3N2)/Victoria/361/2011**								
Kirchenbaum, G. A., et al. (2017) [123]													Samples from: Nachbagauer, R., et al. (2015) = Serum; Samples from: Kirchenbaum, G. A., et al. (2016) = Serum and Blood - PBMCs; Ferret IgG Purification;	Quantitative ferret IgG Igκ and Igλ ELISA; HA-specific IgG Igκ and Igλ ELISA; HA-specific B-cell ELISpot

Hatta, Y., et al. (2018) [83]	male	3-5 month	Triple F Farms or Marshall BioResources	BM2-deficient B(Yam)/Wisconsin/01/2010 LAIV	28 day	BM2-deficient B(Yam)/Wisconsin/01/2010 LAIV	42 day	**A(H1N1)/California/07/2009**	42 day	M2-deficient A(H3N2)/Brisbane/10/2007 LAIV	42 day	**A(H3N2)/Alaska/140/2015**	Nasal wash; Serum	Virus titer; Hemagglutinin Inhibition; HA-specific IgG ELISA
				BM2-deficient B(Yam)/Wisconsin/01/2010 LAIV	28 day	BM2-deficient B(Yam)/Wisconsin/01/2010 LAIV	42 day	**A(H1N1)/California/07/2009**	42 day	Flu-Mist-like isolated A(H3N2)/Brisbane/10/2007 LAIV	42 day	**A(H3N2)/Alaska/140/2015**		
				BM2-deficient B(Vic)/Brisbane/60/2008 LAIV	28 day	BM2-deficient B(Vic)/Brisbane/60/2008 LAIV	42 day	**A(H1N1)/California/07/2009**	84 day	**A(H3N2)/Alaska/140/2015**				
				BM2-deficient B(Vic)/Brisbane/60/2008 LAIV	28 day	BM2-deficient B(Vic)/Brisbane/60/2008 LAIV	42 day	**A(H1N1)/California/07/2009**	84 day	**A(H3N2)/Alaska/140/2015**				
				M2-deficient A(H3N2)/Brisbane/10/2007 LAIV	42 day	**A(H3N2)/Alaska/140/2015**				-				

				Flu-Mist-like isolated A(H3N2)/Brisbane/10/2007 LAIV	42 day	**A(H3N2)/Alaska/140/2015**				-				
				**A(H1N1)/California/07/2009**	90 day	M2-deficient A(H1N1)/California/07/2009 LAIV	42 day	**A(H1N1)/Brisbane/59/2007**						
				**A(H1N1)/California/07/2009**	90 day	Flu-mist-like isolated A(H1N1)/California/07/2009 LAIV	42 day	**A(H1N1)/Brisbane/59/2007**						
				**A(H1N1)/California/07/2009**	132 day	**A(H1N1)/Brisbane/59/2007**								
				M2-deficient A(H1N1)/California/07/2009 LAIV	42 day	**A(H1N1)/Brisbane/59/2007**								
				Flu-mist-like isolated A(H1N1)/California/07/2009 LAIV	42 day	**A(H1N1)/Brisbane/59/2007**								

Francis, M. E., et al. (2019) [18]	Female	5-12 month	Triple F Farms	**A(H1N1)/USSR/90/1977**	67 day	A(H1N1)/California/07/2009; A(H3N2)/Victoria/210/2009; B(Vic)/Brisbane/60/2008; B(Yam)/Florida/04/2006-like QIV (Fluzone: 2015/2016 Northern Hemisphere)	38 day	A(H1N1)/California/07/2009; A(H3N2)/Victoria/210/2009; B(Vic)/Brisbane/60/2008; B(Yam)/Florida/04/2006-like QIV (Fluzone: 2015/2016 Northern Hemisphere)	18 day	**A(H1N1)/California/07/2009**			Nasal wash; Serum; Lung	Virus titer; Histopathology; Hemagglutinin Inhibition; Microneutralization; Virus-specific IgG and IgM ELISA
				**A(H1N1)/USSR/90/1977**	105 day	A(H1N1)/California/07/2009; A(H3N2)/Victoria/210/2009; B(Vic)/Brisbane/60/2008; B(Yam)/Florida/04/2006-like QIV (Fluzone: 2015/2016 Northern Hemisphere)	18 day	**A(H1N1)/California/07/2009**						
				**A(H1N1)/USSR/90/1977**	123 day	**A(H1N1)/California/07/2009**								
Hay, J. A., et al. (2019) [89]	Male and Female	500-1500g	Independent Breeders	**A(H3N2)/Panama/2007/1999**	56 day	**A(H1N1)/Fukushima/141/2006**							Original Data from:	Antibody kinetics modeling;

				**A(H3N2)/Panama/2007/1999**	28 day	A(H1N1)/Solomon Islands/3/2006; A(H3N2)/Brisbane/10/2007; B(Yam)/Brisbane/3/2007 TIV (2008 Southern Hemisphere)	14 day	A(H1N1)/Solomon Islands/3/2006; A(H3N2)/Wisconsin/67/2005; B(Vic)/Malaysia/2506/2004 TIV (2007/2008 Northern Hemisphere)	14 day	**A(H1N1)/Fukushima/141/2006**			Laurie, K. L., et al. (2010)	Biphasic and monophasic antibody waning; exposure-type specific or type non-specific cross-reactivity; antigenic senoirity; priming infection on vaccine response; titre-dependent boosting; Antigenic Distance
				**A(H3N2)/Panama/2007/1999**	28 day	A(H1N1)/Solomon Islands/3/2006; A(H3N2)/Brisbane/10/2007; B(Yam)/Brisbane/3/2007 TIV (2008 Southern Hemisphere) + Adjuvant	14 day	A(H1N1)/Solomon Islands/3/2006; A(H3N2)/Wisconsin/67/2005; B(Vic)/Malaysia/2506/2004 TIV (2007/2008 Northern Hemisphere) + Adjuvant	14 day	**A(H1N1)/Fukushima/141/2006**				

				A(H1N1)/Solomon Islands/3/2006; A(H3N2)/Brisbane/10/2007; B(Yam)/Brisbane/3/2007 TIV (2008 Southern Hemisphere)	14 day	A(H1N1)/Solomon Islands/3/2006; A(H3N2)/Wisconsin/67/2005; B(Vic)/Malaysia/2506/2004 TIV (2007/2008 Northern Hemisphere)	14 day	**A(H1N1)/Fukushima/141/2006**						
				A(H1N1)/Solomon Islands/3/2006; A(H3N2)/Brisbane/10/2007; B(Yam)/Brisbane/3/2007 TIV (2008 Southern Hemisphere) + Adjuvant	14 day	A(H1N1)/Solomon Islands/3/2006; A(H3N2)/Wisconsin/67/2005; B(Vic)/Malaysia/2506/2004 TIV (2007/2008 Northern Hemisphere) + Adjuvant	14 day	**A(H1N1)/Fukushima/141/2006**						
Gooch, K. E., et al. (2019) [88]	Female	930g	Highgate Farm, UK	**A(H1N1)/California/07/2009 low dose challenge model [98]**	28 day	**A(H3N2)/Perth/20/1999 low dose challenge model [98]**							Nasal wash; serum; Blood - PBMCs; Lung - Lung Mononuclear cells	Virus titer; Hemagluttinin Inhibition; IFN-gamma ELISpot; Ferret IFN-gamma ELISA
				**A(H1N1)/California/07/2009 low dose challenge model [98]**	28 day	**A(H1N1)/California/07/2009 low dose challenge model [98]**								

Table 3. Pre-immune ferret studies with only A(H3N2) and Type B influenza in published peer-reviewed literature. Legend: Underlined = Vaccination; Bold = Live infection; TIV = Trivalent inactivated vaccine; LAIV = Live attenuated influenza vaccine; IIV = Inactivated influenza vaccine; VLP = virus-like particle.

Study	Ferret			Influenza Exposures							Samples Taken	Assays conducted
	Sex	Age	Source	1st	Time	2nd	Time	3rd	Time	4th		
Houser, K. V., et al. (2013) [26]	Male	5-8 month	Triple F Farms	**A(H3N2)/Perth/16/2009**	6 week	**A(H3N2)/Perth/16/2009**					Nasal wash; Serum	Virus titer; Hemagglutinin Inhibition; Virus-specific IgG and IgA ELISA
				A(H1N1)/California/07/2009; A(H3N2)/Perth/16/2009; B(Vic)/Brisbane/60/2008 TIV (2011/2012 Northern Hemisphere)	3-5 week	A(H1N1)/California/07/2009; A(H3N2)/Perth/16/2009; B(Vic)/Brisbane/60/2008 TIV (2011/2012 Northern Hemisphere)	3-5 week	**A(H3N2)/Perth/16/2009**				
				**A(H3N2)/Perth/16/2009**	6 week	**A(H3N2)v/Indiana/08/2011**						
				A(H1N1)/California/07/2009; A(H3N2)/Perth/16/2009; B(Vic)/Brisbane/60/2008 TIV (2011/2012 Northern Hemisphere)	3-5 week	A(H1N1)/California/07/2009; A(H3N2)/Perth/16/2009; B(Vic)/Brisbane/60/2008 TIV (2011/2012 Northern Hemisphere)	3-5 week	**A(H3N2)v/Indiana/08/2011**				
				A(H3N2)/Perth/16/2009 IIV	3-5 week	A(H3N2)/Perth/16/2009 IIV	3-5 week	**A(H3N2)v/Indiana/08/2011**				
				A(H3N2)/Beijing/32/1992 IIV	3-5 week	A(H3N2)/Beijing/32/1992 IIV	3-5 week	**A(H3N2)v/Indiana/08/2011**				
				A(H3N2)v/Indiana/08/2011 IIV	3-5 week	A(H3N2)v/Indiana/08/2011 IIV	3-5 week	**A(H3N2)v/Indiana/08/2011**				
				**A(H3N2)/Beijing/32/1992**	6 week	**A(H3N2)v/Indiana/08/2011**						
Kosikova, M., et al. (2018) [79]	Male	15-16 week	Triple F Farms	**A(H3N2)/Switzerland/9715293/2013**	2 week	**A(H3N2)/Hong Kong/4801/2014**					Serum; Purified IgG	Virus titer; Hemagglutinin Inhibition; Neutralization; HA-specific IgG ELISA; HA-specific IgG Avidity;
				**A(H3N2)/Texas/50/2012**	2 week	**A(H3N2)/Switzerland/9715293/2013**	2 week	**A(H3N2)/Hong Kong/4801/2014**				
				**A(H3N2)/Uruguay/716/2007X175C**	2 week	**A(H3N2)/Texas/50/2012**	2 week	**A(H3N2)/Switzerland/9715293/2013**	2 week	**A(H3N2)/Hong Kong/4801/2014**		

												Antigenic cartography
Allen, J. D., et al. (2019) [37]	Spayed Female	6-12 month	Triple F Farms	**A(H3N2)/Panama/20/1999**	84 day	T-6 COBRA HA VLP + Adjuvant	84 day	T-6 COBRA HA VLP + Adjuvant			Serum	Hemagglutinin Inhibition; Focal Reduction Neutralization
				**A(H3N2)/Panama/20/1999**	84 day	T-7 COBRA HA VLP + Adjuvant	84 day	T-7 COBRA HA VLP + Adjuvant				
				**A(H3N2)/Panama/20/1999**	84 day	T-10 COBRA HA VLP + Adjuvant	84 day	T-10 COBRA HA VLP + Adjuvant				
				**A(H3N2)/Panama/20/1999**	84 day	T-11 COBRA HA VLP + Adjuvant	84 day	T-11 COBRA HA VLP + Adjuvant				
				**A(H3N2)/Panama/20/1999**	84 day	A(H3N2)/Wisconsin/67/2005 HA VLP + Adjuvant	84 day	A(H3N2)/Wisconsin/67/2005 HA VLP + Adjuvant				
				**A(H3N2)/Panama/20/1999**	84 day	A(H3N2)/Texas/50/2012 HA VLP + Adjuvant	84 day	A(H3N2)/Texas/50/2012 HA VLP + Adjuvant				

Table 4. Pre-immune ferret studies with A(H5N1) influenza in published peer-reviewed literature. Legend: Underlined = Vaccination; Bold = Live infection; TIV = Trivalent inactivated vaccine; LAIV = Live attenuated influenza vaccine; IIV = Inactivated influenza vaccine; VLP = virus-like particle.

Study	Ferret			Influenza Exposures			Samples Taken	Assays conducted
	Sex	Age	Source	1st	Time	2nd		
Cheng, X., et al. (2009) [92]	Unknown	7-10 week	Triple F Farms	A(H1N1)/New Caledonia/20/1999; A(H3N2)/California/7/2004; B(Yam)/Jilin/20/2003 LAIV (FluMist: 2005/2006 Northern Hemisphere)	4-6 week	A(H5N1)/Hong Kong/213/2003 LAIV	Nasal wash; Serum; Blood - PBMC; Paratracheal lymph nodes	Virus Titer; Hemagglutinin Inhibition; Neutralization; HA-specific IgG ELISA; Virus and HA-specific B-cell ELISpot
				A(H1N1)/New Caledonia/20/1999; A(H3N2)/California/7/2004; B(Yam)/Jilin/20/2003 LAIV (FluMist: 2005/2006 Northern Hemisphere)	4-6 week	A(H5N1)/Vietnam/1203/2004 LAIV		
				A(H3N2)/California/7/2004 LAIV	42 day	A(H5N1)/Hong Kong/213/2003 LAIV		
				A(H5N1)/Hong Kong/213/2003 LAIV	42 day	A(H5N1)/Hong Kong/213/2003 LAIV		
				A(H1N1)/New Caledonia/20/1999 LAIV	42 day	A(H5N1)/Hong Kong/213/2003 LAIV		
				A(H1N1)/New Caledonia/20/1999 LAIV	42 day	A(H5N1)/Vietnam/1203/2004 LAIV		

				A(H1)/New Caledonia/20/1999:A(N2)/Wyoming/03/2003 LAIV	5 week	A(H5N1)/Vietnam/1203/2004 LAIV		
				A(H5N1)/Vietnam/1203/2004 LAIV	42 day	A(H5N1)/Vietnam/1203/2004 LAIV		

© 2020 by the authors. Submitted for possible open access publication under the terms and conditions of the Creative Commons Attribution (CC BY) license (http://creativecommons.org/licenses/by/4.0/).
